# Elevated CHCHD4 orchestrates mitochondrial oxidative phosphorylation to disturb hypoxic pulmonary hypertension

**DOI:** 10.1186/s12967-023-04268-3

**Published:** 2023-07-12

**Authors:** Yu Wang, Zhenyu Zeng, Zhaoxiang Zeng, Guojun Chu, Xinghua Shan

**Affiliations:** 1 Department of Cardiology, Changhai Hospital, Navy Medical University, 168 Changhai Road, Shanghai, 200433 People’s Republic of China; 2 Department of Vascular Surgery, Changhai Hospital, Navy Medical University, Shanghai, People’s Republic of China

**Keywords:** CHCHD4, PASMC, Mitochondria, SAM50, PAH

## Abstract

**Background:**

Pulmonary arterial hypertension (PAH) is a highly prevalent cardiopulmonary disorder characterized by vascular remodeling and increased resistance in pulmonary artery. Mitochondrial coiled–coil–helix–coiled–coil–helix domain (CHCHD)-containing proteins have various important pathophysiological roles. However, the functional roles of CHCHD proteins in hypoxic PAH is still ambiguous. Here, we aimed to investigate the role of CHCHD4 in hypoxic PAH and provide new insight into the mechanism driving the development of PAH.

**Methods:**

Serotype 1 adeno‐associated viral vector (AAV) carrying Chchd4 was intratracheally injected to overexpress CHCHD4 in Sprague Dawley (SD) rats. The Normoxia groups of animals were housed at 21% O_2_. Hypoxia groups were housed at 10% O_2_, for 8 h/day for 4 consecutive weeks. Hemodynamic and histological characteristics are investigated in PAH. Primary pulmonary artery smooth muscle cells of rats (PASMCs) are used to assess how CHCHD4 affects proliferation and migration.

**Results:**

We found CHCHD4 was significantly downregulated among CHCHD proteins in hypoxic PASMCs and lung tissues from hypoxic PAH rats. AAV1-induced CHCHD4 elevation conspicuously alleviates vascular remodeling and pulmonary artery resistance, and orchestrates mitochondrial oxidative phosphorylation in PASMCs. Moreover, we found overexpression of CHCHD4 impeded proliferation and migration of PASMCs. Mechanistically, through lung tissues bulk RNA-sequencing (RNA-seq), we further identified CHCHD4 modulated mitochondrial dynamics by directly interacting with SAM50, a barrel protein on mitochondrial outer membrane surface. Furthermore, knockdown of SAM50 reversed the biological effects of CHCHD4 overexpression in isolated PASMCs.

**Conclusions:**

Collectively, our data demonstrated that CHCHD4 elevation orchestrates mitochondrial oxidative phosphorylation and antagonizes aberrant PASMC cell growth and migration, thereby disturbing hypoxic PAH, which could serve as a promising therapeutic target for PAH treatment.

**Graphical Abstract:**

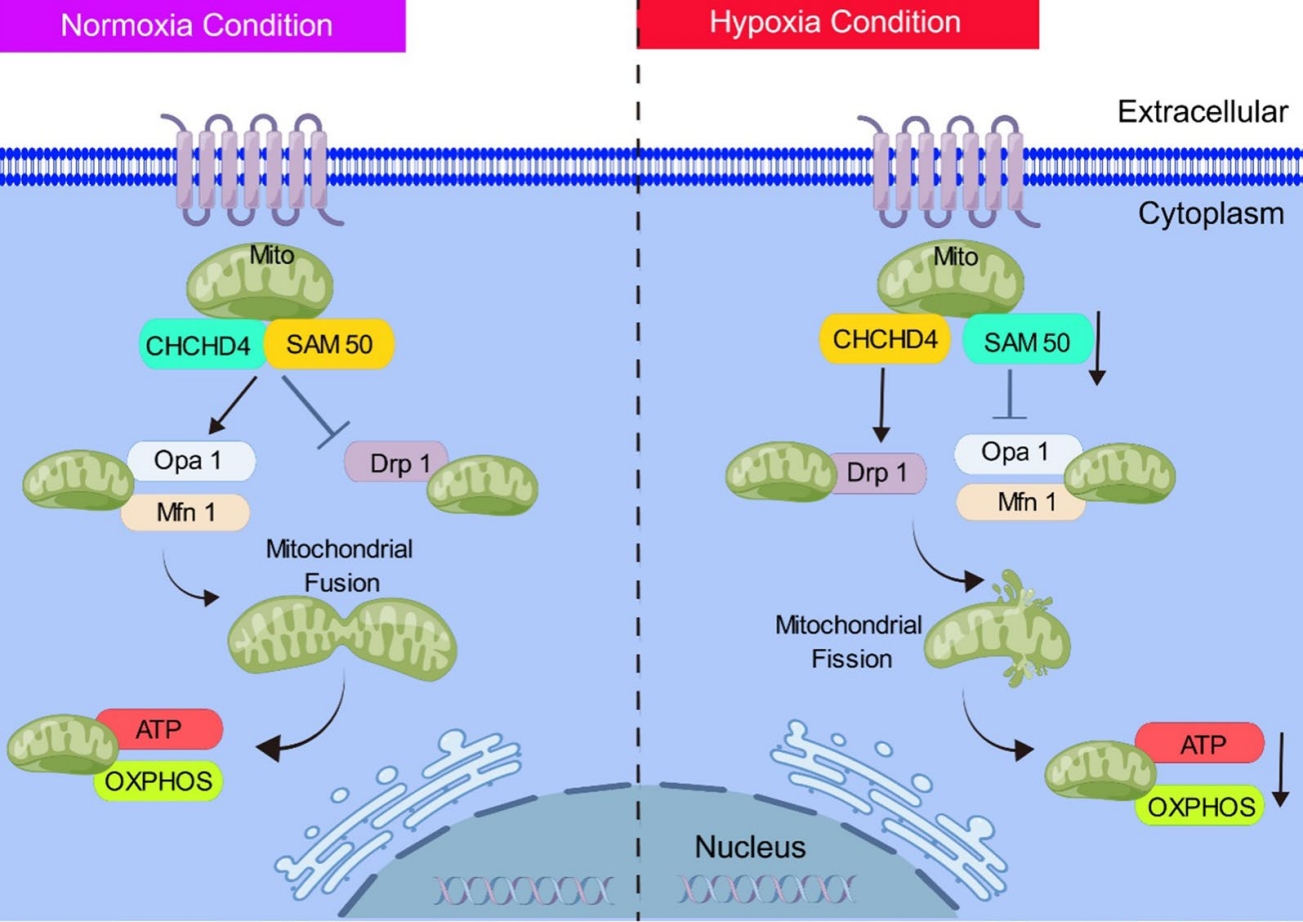

**Supplementary Information:**

The online version contains supplementary material available at 10.1186/s12967-023-04268-3.

## Introduction

As a highly prevalent cardiopulmonary disorder, pulmonary arterial hypertension (PAH) is characterized with diffused vascular remodeling and pulmonary artery resistance, resulting in subsequent right ventricular dysfunction [1, 2]. Dysfunctional pulmonary arterial smooth muscle cells were manifested with abnormal proliferation and migration of PASMCs, together driving progressive remodeling of peripheral pulmonary arteries and augmented right ventricular afterload [3, 4]. However, the cellular and molecular mechanism modulating hypoxic PAH remains undefined. In addition, Metabolic reprogramming serves as a crucial driver in PAH, including hyper-glycolytic reprogramming, aberrant iron handling, insulin insensitivity, and defective sphingosine metabolism [5–8], however, whether and how mitochondrial dynamics regulates hypoxic PAH remain incompletely understood.

The coiled–coil–helix–coiled–coil–helix domain (CHCHD)-containing proteins are identified as a kind of conserved nucleus-encoded small mitochondrial proteins [9], among which have various important pathophysiological roles. Recently, CHCHD2 has been suggested to be associated with motor dysfunction and pathogenesis of Parkinson's disease (PD) [10, 11], particularly in mitochondrial homeostasis [12, 13]. Specifically, hepatocyte CHCHD2 contributed to liver fibrosis in nonalcoholic fatty liver disease (NAFLD) [14], whereas CHCHD3 was found to improve mitochondrial function via increasing mitochondrial ROS and promoting ferroptosis, by which modulated tumorigenesis [15]. In line with CHCHD2, CHCHD10 has been verified to be involved in multiple neurological alterations including Parkinson’s disease (PD), frontotemporal dementia (FTD), as well as Alzheimer's disease (AD) [13, 16]. CHCHD10 also serves as a mitochondrial modulator in adipose browning [17] and thermogenesis of adipocytes [18]. Notably, CHCHD4 was found to be widely expressed in a variety of tissues and multiple human cell types [19, 20] and regulated various cellular and/or mitochondrial functions, including maintaining mitochondrial DNA (mtDNA) homeostasis, proteins assembly, mitochondria oxidative phosphorylation (OXPHOS), and cellular oxygenation [20]. Specifically, increasing evidence indicates that CHCHD4 play important roles in regulating mitochondrial respiratory chain, which in turn affects tumor proliferation and EMT-related phenotypes, cellular oxygen consumption rate and metabolism, as well as energy homeostasis [21–24]. However, the role and exact molecular mechanism of CHCHD4 involved in hypoxic PAH remain unclear.

The stability and quality control of mitochondrial network, along with the support of mitochondrial OXPHOS, were maintained through mitochondrial dynamics, including mitochondrial fusion and fission [25, 26]. Various diseases are linked with abnormal mitochondrial dynamics, which contributed to mitochondrial dysfunction and impaired cell proliferation [27, 28]. Thus, there will be great clinical potential to target mitochondrial dynamics to treat a variety of human diseases [29], including PAH. In current study, we demonstrated that CHCHD4 elevation orchestrates mitochondrial oxidative phosphorylation and antagonizes aberrant proliferation and migration of PASMCs, thereby disturbing hypoxic PAH, which could be a novel target for PAH treatment.

## Materials and methods

### Antibodies

All antibodies utilized in this study: anti-CHCHD4 (Proteintech, #21090-1-AP); anti-β-Actin (Proteintech, #81115-1-RR); anti-α-SMA (CST, #19245S); anti-citrate synthetase (Abcam, # ab129095); anti-Vimentin (Abcam, #ab92547); anti-PCNA (Santa Cruz, #sc-56); anti-Ki67 (Abcam, #ab15580); anti-Opa1 (Santa Cruz, # sc-393296); anti-Drp1 (Santa Cruz, # sc-271583); anti-Mfn1 (Santa Cruz, #sc-166644); anti-SAM50 (Abcam, #ab246987); anti-DYKDDDDK Tag (CST, #14793); anti-HA (CST, #3724).

### Animals and treatment

Sprague Dawley (SD) rats (100–150 g, 1-month‐old) were acquired from the Institute of Laboratory Animal Science, Chinese Academy of Medical Sciences (Beijing, China). All animals were randomly divided into four groups: Normoxia + AAV-CTR (n = 9), Hypoxia + AAV-CTR (n = 9), Normoxia + AAV-CHCHD4 (n = 9) and Hypoxia + AAV‐CHCHD4 (n = 9). All rodents were continuously housed in pathogen‐free (SPF) environment in Model Animal Center of Navy Medical University. Serotype 1 AAV carrying Chchd4 was intratracheally injected at a concentration of at least 1.0 × 10 [12] vector genomes (vg)/ml three days ahead of Normal or PAH model, and the dose of the injected vectors was 100 μL in each animal. The AAV-CTR vector was used as control. The transduction efficiency was verified by western blot after 2 weeks of viral vector expression. Animals in Normoxia group were kept at 21% O_2_, whereas hypoxia group were housed at 10% O_2_, for 8 h/day for 4 consecutive weeks. The efficiency of CHCHD4 overexpression lung artery tissues were verified using immunoblotting (Additional file 1: Fig. S2A).

### Hemodynamic measurements

After the Normoxia or hypoxia exposure 28th day, before hemodynamic examination animals from each group were subjected to sodium pentobarbital (60 mg/kg) injection for anaesthetization. Especially, right ventricular systolic pressure (RVSP) was directly monitored by right heart catheterization using a multichannel physiological recorder monitoring equipment (BL-420 F). In addition, the whole heart was harvested for weight analysis. Importantly, the right ventricle (RV) was isolated from the rest of the heart (left ventricular (LV) and interventricular septum (S)), and the weights were then recorded. Last, RV hypertrophy was assessed by ratio of RV to the LV + S [RV/(LV + S)].

### Cell culture and treatment

Primary PASMCs, obtained from Procell Life Technology, were cultured in DMEM‐F12 medium supplemented with 10% fetal bovine serum (FBS). Hypoxic cell model was generated by distinguish atmosphere in culture chamber. Briefly, cells from Normoxic group were cultured in condition of 21% O_2_, 74% N_2_, and 5% CO_2_, whereas hypoxic cells were held under 2% O_2_, 93% N_2_, and 5% CO_2_ at 37 °C.

### Histological analysis

The lung tissue was sequentially soaked in 4% cell fixative solution and embedded with paraffin. Subsequently, it was cut into 5.0 μm sections used for Hematoxylin and Eosin (H&E) staining. Images were captured by optical microscope (Olympus, Japan), then the ratio of pulmonary arterial medial thickness to total vessel size (media/CSA) was determined by Image J software.

### Immunofluorescence staining

For IF assay in vivo, paraffin-embedded lung tissue of rats was incubated with primary antibody including anti-CHCHD4, anti-α-SMA and anti-Ki67 overnight at 4 °C, subsequently, Alexa Fluor™ 568 (A-11011, Invitrogen) or Alexa Fluor™ 488 (A-11001, Invitrogen) was incubated. Next, we added fluorescent mounting medium with DAPI (D21490, Invitrogen). For IF assay in vitro, PASMCs climbing sheets were incubated with primary antibody with anti-CHCHD4, anti-Vimentin and anti-α-SMA following by incubation with Alexa Fluor™ 488, Alexa Fluor™ 568 and added fluorescent mounting medium with DAPI. For mitochondrial morphology measurement, Mito Tracker was used to present mitochondria and the mean mitochondrial length was determined in each PASMCs using Image J software (as showed in Fig. 5E and 7A with white arow).

### Lentivirus transduction and siRNA transfections

PASMCs were transduced with lentivirus packaging with empty plasmid (len-CTR) or plasmid vector containing cDNA of Chchd4 (len-CHCHD4) for 48 h. Small interfering RNA (siRNA) targeting SAM50 or CHCHD4 was incubated with PASMCs together with lipo6000 ™ for 48 h. Western blot were utilized to verify the overexpression of CHCHD4 and knockdown of SAM50 or CHCHD4 (Additional file 1: Fig. S2C). The sequence for SAM50 as followed: 5′-GATAATCGATGACC TTTTTGGAAA-3′; and control sequence 5′-AG CTTTTCC AATTGAATGACCGGG-3′. The sequence for CHCHD4 as followed: 5′-GCAUGGAU UGAUACUGCCATT-3′; and control sequence 5′-GGG CAAAAUCGAUAAUUCU-3′

### Seahorse experiment

Mitochondrial oxygen consumption rate (OCR) of PASMCs was evaluated by the Seahorse XFe24, following manufacturer’s guidelines (Agilent Technologies). In brief, NRCMs were treated with Oligomycin (1.0 μM), followed by 0.25 μM trifluoromethoxy carbonyl cyanide phenylhydrazone (FCCP), and 0.50 μM Rotenone and Antimycin A. Next, OCR was recorded by Seahorse Wave [30]. Glycolytic stress test was measured by extracellular acidification rate (ECAR), this entailed exposure of the PASMCs to glucose (10 mM), Oligomycin (1.0 μM), and 2-deoxy-glucose (50 mM).

### Assessment of ATP content

ATP assay kit (S0026, Beyotime, China) was utilized in ATP detection, according to the manufacturer instructions. In brief, pulmonary artery tissues were homogenized by ATP detection lysis buffer, the supernatant was utilized to evaluate ATP content using Centro XS3 LB 960 luminometer (Berthold Technologies, Germany) after centrifugation, and the ATP concentration was measured by ATP standard curve.

### Transmission electron microscope

The lung tissues isolated from indicated animals were fixed with the electron microscope fixative (0.1 mol/L sodium cacodylate solution) at 4 ℃ for 24 h. Subsequently, the electron microscope buffer (0.1 mol/L of sodium cacodylate buffer) was used for specimens washing, and1% osmium tetroxide was utilized for secondary fixation for 2 h. Afterward, the lung specimens were gradiently dehydrated before immersed and embedded by Epon812. Images of the specimens were taken by Transmission electron microscope (TEM) in a thickness of 60 nm. For mitochondrial density, the total mitochondrial area and mitochondria with missing cristae (disarrayed cristae and a reduced electron density in the matrix) were determined in each field and divided by the total tissue area using Image J software.

### RNA-sequencing analysis

Lung tissue homogenate (n = 3 per group) was harvested for RNA-sequencing (RNA-seq) analysis. The RNA isolated from indicated rats was analyzed by Cloundseq mRNA enrichment kit in Illumina Sequencer. The differential gene expression between PAH model rats injected with CHCHD4-O/E or CTR was detected by Cuffdiff software, differentially expressed genes (DEGs) were then identified by the following criteria: (1) fold-change (FC) log |FC|≥ 1.0 with P ≤ 0.05; (2) fragments per kilobase million (FPKM) value ≥ 0.1.

### Western blotting

In addition to lung tissues homogenates, mitochondria were isolated by using Tissue Mitochondria Isolation Kit (C3606, Beyotime, China), according to the manufacturer instructions. Cytosolic and nuclear proteins were simultaneously obtained by Nuclear and Cytoplasmic Protein Extraction Kit (P0027, Beyotime, China). Proteins extracted from cytosolic, mitochondrial and nuclear fractions were separated through electrophoresis with 10% SDS-PAGE gels, subsequently protein components were transferred onto the PVDF membrane and blocked with 5% skimmed milk for 1 h. The membrane was incubated overnight at 4 °C with primary antibody, followed by incubation with HRP-conjugated secondary antibody (1: 10,000) at room temperature for 1 h. Finally, the membrane was scanned by Image Quant system after soaking with ECL reagents. Notably, the densitometry analysis of protein bands was obtained by the Image Lab software, and normalized against β-Actin (tissue homogenates and cytosolic fractions), Citrate synthase (mitochondrial fractions) or PCNA (nuclear fractions). For immunoprecipitation assay. Before incubated at 4 ℃ overnight, tissue homogenate or cell whole lysates were blended in GFP-binder or antibody-coupled protein G-Sepharose. The non-specific binding proteins were subsequently elution, and immunoprecipitants were separated in SDS gels.

### Gene expression measurement by RT-qPCR analysis

Total RNA was extracted from PASMCs or lung tissues using TRIzol reagent (Invitrogen), and qPCR was performed using Light Cycler 480 SYBR Green Master Mix with 43 reaction cycles. Gene expression of Chchd4 was normalized to reference gene GAPDH, and the primer sequences were listed here: Chchd4 F 5′-ACAACGAGAAGGTGTAGCCG-3′; R 5′-AGAAAGTCCCATGGTGAGTGG-3′. Gapdh F 5′-CGCATGAACACTCT GGAGATG-3′; R 5′-TGTGAGGGACTCTGGT CTTTGT-3′.

### Transwell assays

To evaluate cell migration, transfected cells were seeded into the upper chamber of a 24-well transwell plate under starvation pretreatment, while SMCGM with 5% FBS was added to the lower chamber. PASMCs were allowed to migrate continuously for 24 h at 37 ℃, followed by staining with 0.1% crystal violet (30 min, RT) and fixation in 4% paraformaldehyde. Cell counting was performed using Zeiss Axio Vert A1 microscope, and cell images were recorded from six random fields by using Image J software.

### Cell proliferation experiment

Cell proliferation was measured by CCK-8 assay (Dojindo) at indicated time points following manufacturer’s instructions. The absorbance was recorded at 450 nm was detected by microplate reader (Biotek, USA).

### Wound healing analysis

PASMCs were cultured until reaching confluency, then plate was scratched using 200 μL pipette tip. These cells were cultured with serum free medium under Normoxia or hypoxia condition for additional 24 h. Images were taken at 0 and 24 h using a Zeiss Axio Vert. A1 microscope. The wound area was evaluated and the percentage of the wound healing was calculated by Image J software.

### Statistical analysis

All data were shown as Mean ± SEM. Unpaired two-tailed Student’s t-test was conducted to compare 2 groups, while one-way ANOVA followed by Dunnett's multiple comparisons and two-way ANOVA followed by Bonferroni's multiple comparisons were used to compare multiple groups with various treatments and time points. Paired *t*-test was employed to asses OCR and ECAR. GraphPad Prism 8.0 was used for all tests, and p < 0.05 was considered statistically significant.

## Results

### CHCHD4 is downregulated in hypoxic PAH

**Fig. 1:**
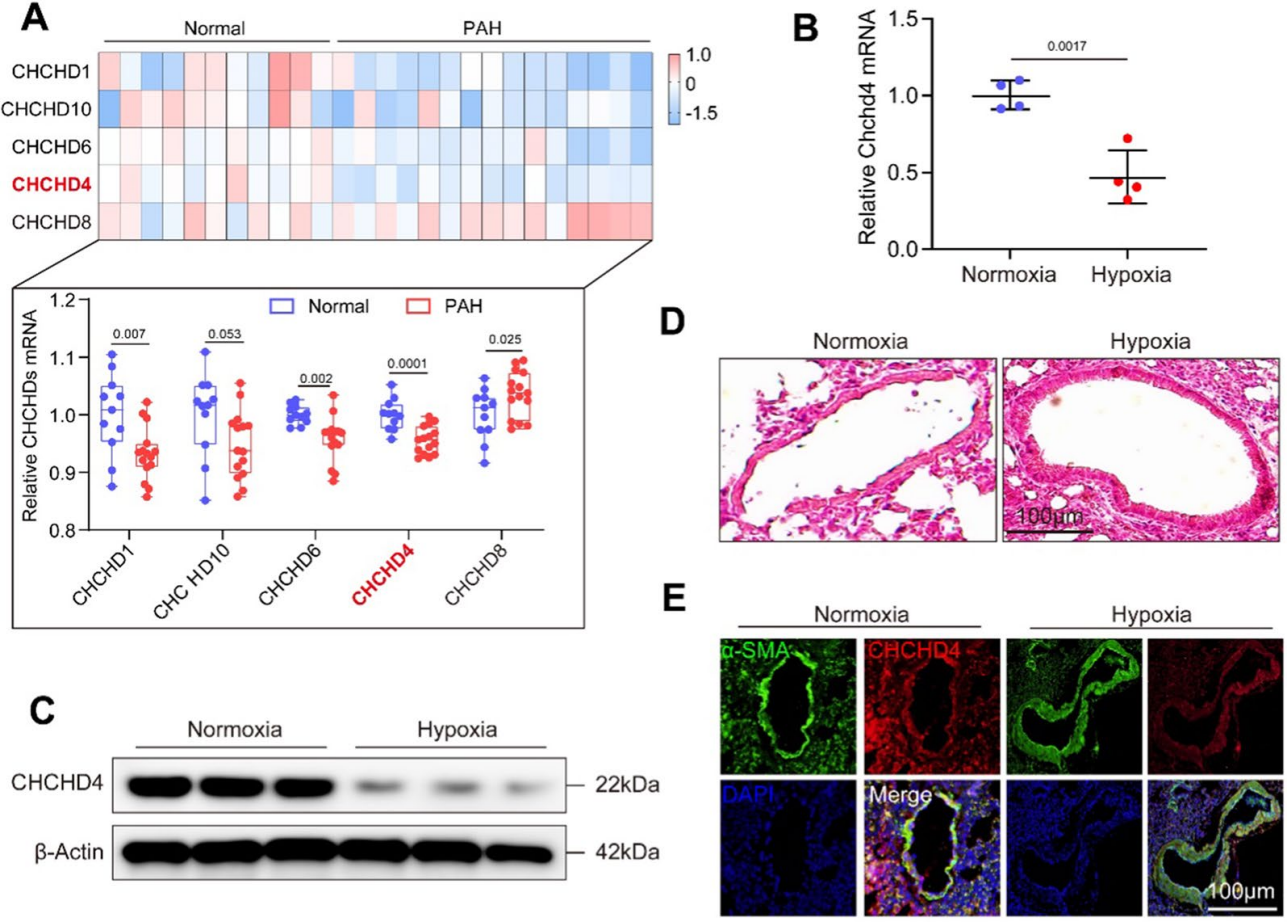
CHCHD4 is identified as a regulator of PAH. **A** Differential expression CHCHD protein among CHCHD protein family in PAH patients (GSE113439). **B** Quantitative real time PCR (qPCR) analysis of Chchd4 transcription in lung tissues from indicated animals (n = 4). **C** Representative immunoblotting of CHCHD4 in lung tissues (n = 6), Normalized to β-Actin. **D** Representative HE staining verified successful PAH model. **E** Representative immunofluorescence of α-SMA (green), CHCHD4 (red) and DAPI (blue) in lung tissues from indicated group. Data are shown as the mean ± SEM. P value is showed in each figure

**Fig. 2:**
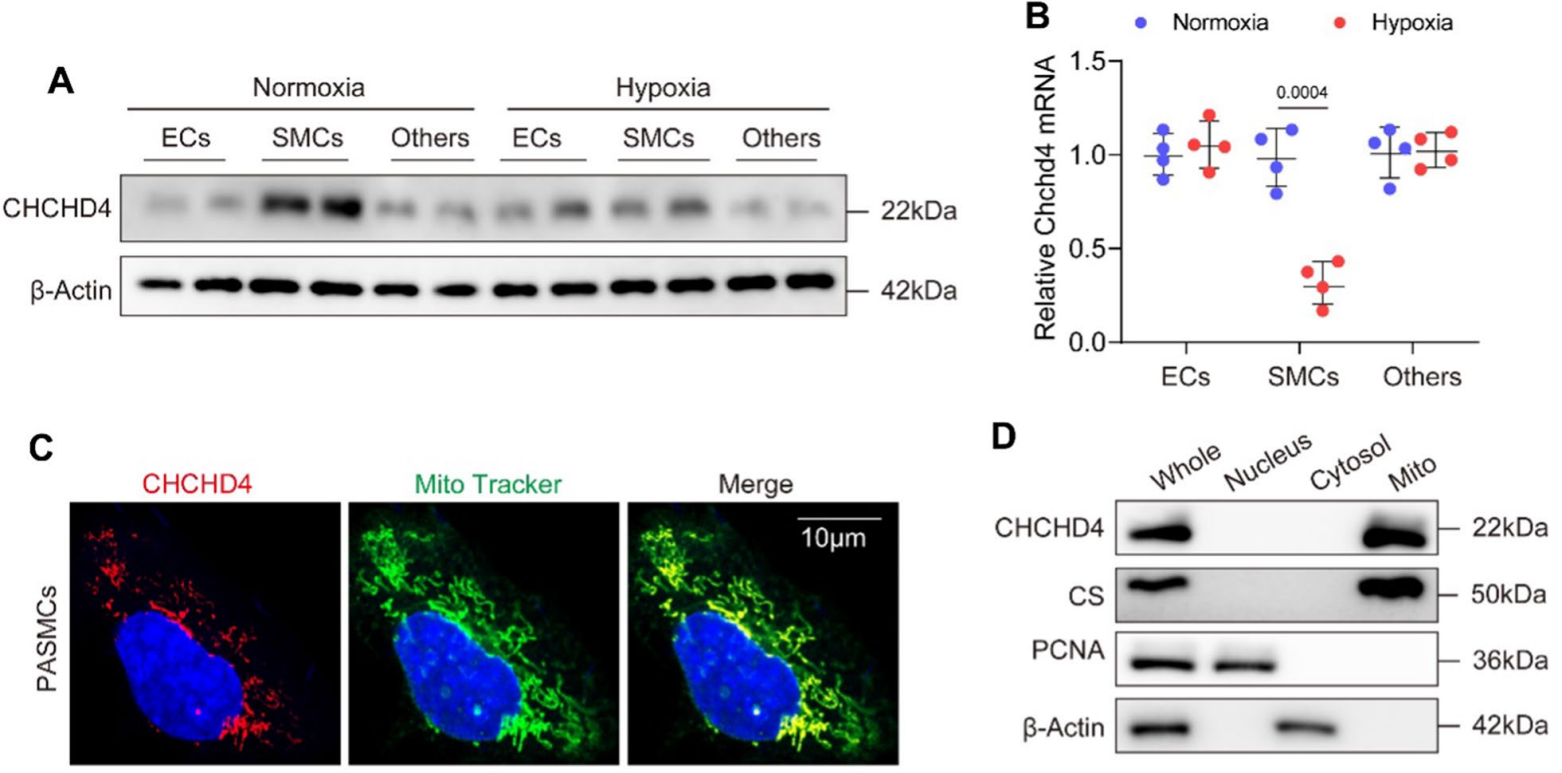
CHCHD4 is downregulated in PASMCs and located in mitochondria. **A** Representative immunoblotting of CHCHD4 in isolated cells (n = 4), Normalized to β-Actin. **B** qPCR analysis of Chchd4 transcription in various cell types (n = 4). **C** Representative immunofluorescence of CHCHD4 and Mito tracker green in PASMCs. **D** Representative immunoblotting of CHCHD4 in cell components, Normalized to citrate synthase (CS) in mitochondria, PCNA in nucleus and β-Actin in cytoplasm. Data are shown as the mean ± SEM. P value is showed in each figure

To identify the differential expression CHCHD protein among CHCHD protein family, we first characterized gene expression profiling using microarray results from GEO database (https://www.ncbi.nlm.nih.gov/gds/, GSE113439) [31]. We observed that most mitochondrial CHCHD proteins were integrally decreased under hypoxic condition, compared to Normoxia, except for CHCHD8. Notably, gene expression of CHCHD4 in the lung was markedly downregulated from PAH patients, when compared to healthy controls (Fig. 1A). Next, we generated hypoxic PAH in SD rats (Fig. 1D and Additional file 1: Fig. S1A). Consistently, reduced CHCHD4 transcription and protein expression were exhibited in lungs of animals from hypoxic PAH group, in contrast to normoxia controls (Fig. 1B, C and Additional file 1: Fig. S1B). To test whether CHCHD4 contributes to aberrant PASMCs proliferation, we stained lung tissues from hypoxic PAH or Normoxia rat with CHCHD4 and α-SMA. The results showed a decrease in CHCHD4 expression in hypoxia group when comparing to those from Normoxia group (Fig. 1E and Additional file 1: Fig. S1C). Consistently, we stained lung tissues from hypoxic PAH or Normoxia rat with CHCHD4 and Vimentin and found a decrease in CHCHD4 expression in hypoxia group (Additional file 1: Fig. S1D). Next, to investigate the specific cell type, primary rat VSMCs, endothelial cells and other cells were isolated from adult SD rats (Additional file 1: Fig. S2B). Consequently, we found that CHCHD4 was mainly expressed in SMCs instead of endothelial cells (ECs) or other cells (epithelial cells, monocyte and lymphocytes), while CHCHD4 protein was conspicuously downregulated in response to hypoxic stress (Fig. 2A). In line with the data of immunoblotting, reduced CHCHD4 mRNA levels were also exhibited according to the results of real-time quantitative PCR (Fig. 2B). To verify the subcellular localization of CHCHD4 in PASMCs, we assess the expression of CHCHD4 in each component of purified PASMCs by immunofluorescence and immunoblotting. Expectedly, CHCHD4 proteins were almost enriched in mitochondria (Fig. 2C), instead of nuclear and cytoplasmic components (Fig. 2C, D). Overall, these results indicate that mitochondrial CHCHD4 is substantially downregulated in pulmonary artery of hypoxia-induced PAH and is associated with PASMCs.

### Elevated CHCHD4 alleviates hypoxia-triggered PAH induced

**Fig. 3:**
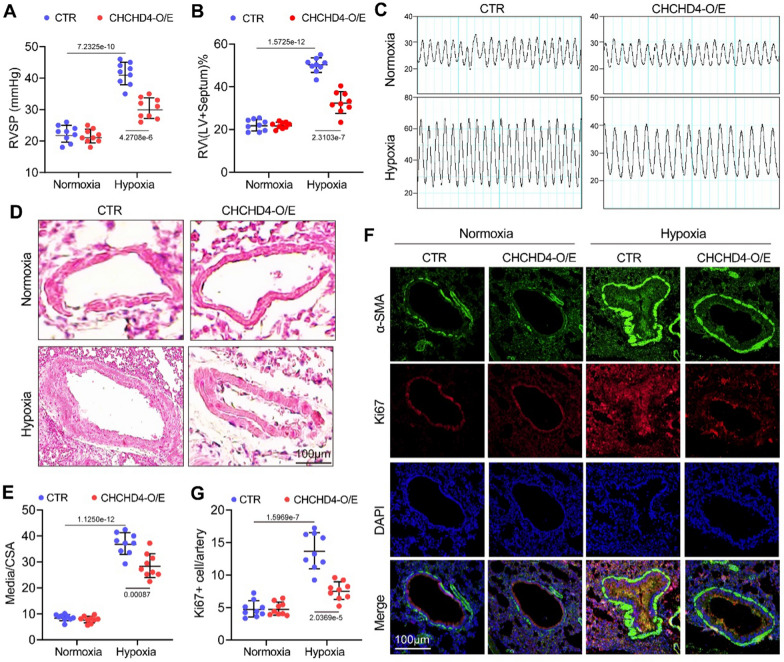
CHCHD4 attenuates hypoxic PAH. Hemodynamic examination was analyzed after the Normoxia or hypoxia exposure 28th day and then animals sacrificed. **A** The RVSP of the Normoxia and hypoxia rats with or without AAV-CHCHD4 treatment (n = 9). **B** Summary data of the right ventricle/(left ventricle + septum) weight ratio (n = 9). **C** Representative images of RVSP measurements in Normoxia and hypoxia rats with or without AAV-CHCHD4 treatment (n = 9). **D** H&E staining of pulmonary arteries in lung tissues in indicated animals. **E** Quantification of ratio of pulmonary arterial medial thickness to total vessel size (media/CSA) in each group (n = 9). **F** Double immunofluorescence staining of α-SMA (green) and Ki67 (red) in pulmonary arteries of the Normoxia and hypoxia rats with or without AAV-CHCHD4 treatment. **G** Quantification of Ki67 positive cells in pulmonary arteries (n = 9). Data are shown as the mean ± SEM. P value is showed in each figure

To investigate the functional roles of CHCHD4 on hypoxia-mediated PAH, AAV1 carrying Chchd4 was utilized to overexpress CHCHD4 in PAH rats. As expected, although exogenous CHCHD4 showed no effect on RVSP in Normoxia animals, increased CHCHD4 levels notably reduced RVSP under hypoxia condition (Fig. 3A, C). Moreover, administration of AAV1-CHCHD4 markedly reduced the increased RV/(LV + S) ratio under hypoxia condition, despite AAV1-CHCHD4 injection had limited influence on RV/(LV + S) ratio in Normoxia-treated rats (Fig. 3B). Consistently, histological measurements showed that pulmonary arterial medial wall thickness was notably increased, which could be suppressed by upregulating CHCHD4 under hypoxia condition (Fig. 3D, E). In addition, the data of immunofluorescence showed that overexpression of CHCHD4 suppressed PASMCs proliferation in hypoxia-treated pulmonary artery, as identified by decreased Ki67 signal in medial wall (Fig. 3F, G). These data demonstrated that elevated CHCHD4 ameliorated the development of hypoxia-triggered PAH.

### CHCHD4 modulates hypoxia-induced proliferation and migration in PASMCs

**Fig. 4:**
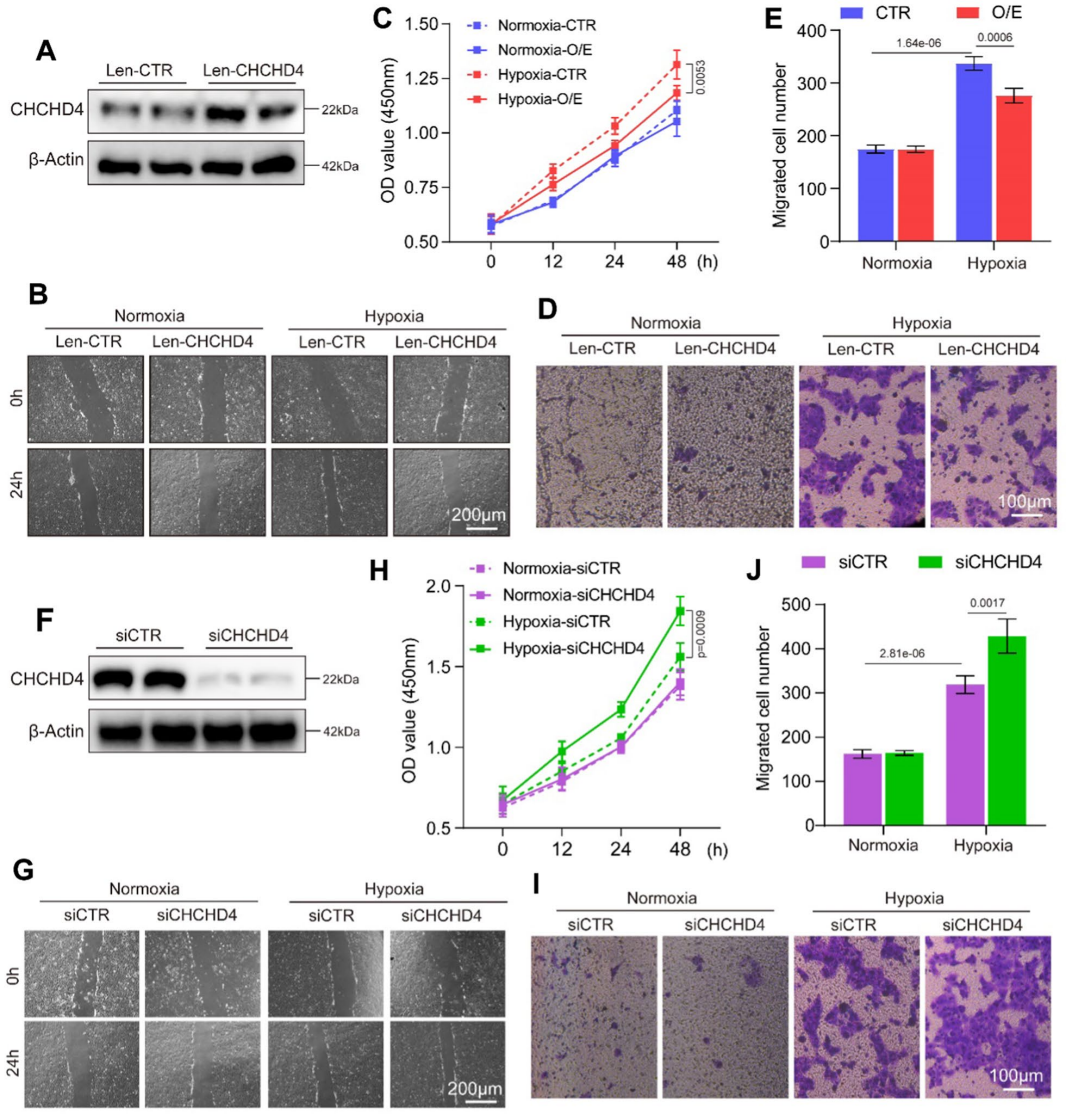
CHCHD4 affects hypoxia-induced proliferation and migration in PASMCs. **A** Representative immunoblotting of CHCHD4 in PASMCs incubated with len-CTR or len-CHCHD4 (n = 4). **B** Wound healing assay of PASMCs in each group (n = 3 independent experiments). **C** Results of the CCK8 cell viability assay of PASMCs in each group (n = 3 independent experiments). **D**, **E** Representative images and summary data of the Transwell assay, which indicated the migration ability of PASMCs in indicated groups (n = 3 independent experiments). **F** Representative immunoblotting of CHCHD4 in PASMCs incubated with siCTR or siCHCHD4 (n = 4). **G** Wound healing assay of PASMCs in each group (n = 3 independent experiments). **H** Results of the CCK8 cell viability assay of PASMCs in each group (n = 3 independent experiments). **I**, **J** Representative images and summary data of the Transwell assay, which indicated the migration ability of PASMCs in indicated groups (n = 3 independent experiments). Data are shown as the mean ± SEM. P value is showed in each figure

To evaluate how CHCHD4 modulates proliferation and migration of SMCs, CHCHD4 was overexpressed in PASMCs through transduction using lentivirus vector encoding Chchd4 mRNA followed by hypoxic induction. As shown in Fig. 4A, CHCHD4 gene expression was markedly increased after transduction. Further analysis showed that PASMCs exhibited increased proliferation and migration under hypoxic conditions. Importantly, such effects were mitigated by CHCHD4 overexpression. (Fig. 4B–E and Additional file 1: Fig. S3A). Conversely, knockdown of CHCHD4 presented opposite results. Immunoblotting data showed that siRNA targeting CHCHD4 significantly disrupted the CHCHD4 protein expression in PASMCs (Fig. 4F). Importantly, knockdown of CHCHD4 could further promote PASMC proliferation and migration during hypoxia (Fig. 4G–J and Additional file 1: Fig. S3B). Overall, these findings suggest indicated that CHCHD4 plays important roles in regulating PASMC proliferation and migration under hypoxic conditions.

### CHCHD4 antagonizes mitochondrial defects and dysfunction

**Fig. 5:**
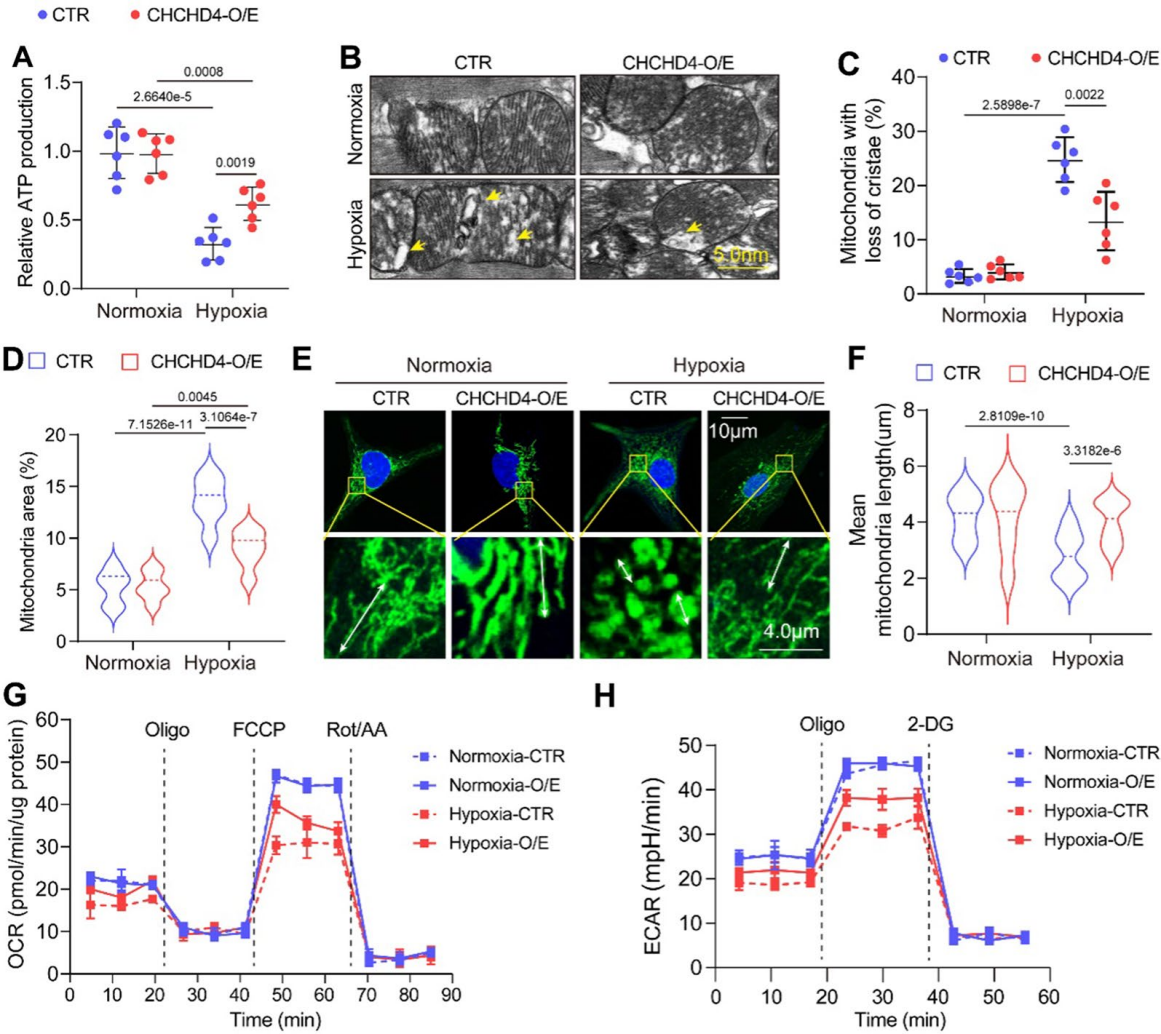
Overexpression of CHCHD4 improves hypoxia-induced mitochondrial dysfunction. **A** Quantification of ATP in lung tissues from Normoxia or Hypoxia treated animals with or without AAV-CHCHD4 injection (n = 6) in vivo. **B** Representative EM image showing disarrayed cristae and reduced electron density of the matrix in the mitochondria in vivo. The yellow arrows showed the mitochondria with missing cristae (disarrayed cristae and a reduced electron density in the matrix). **C** Quantification of the percent of damaged mitochondria (n = 6) in vivo. **D** Quantification of mitochondria area%, which refers to the ratio of mitochondrial area to image area (n = 264–381) in vivo. **E**, **F** Representative confocal images of Mitochondrial Tracker in indicated group and quantification of the mitochondrial mean length (n = 62–115 PASMCs) in vitro. White arow indicated the mitochondrial length. **G** Real-time monitoring the oxygen consumption rate (OCR) in PASMCs (n = 6) in vitro. Oligo: oligomycin; FCCP: trifluoromethoxy carbonyl cyanide phenylhydrazone; Rot/AA: Rotenone/AntimycinA. **H** Real-time monitoring the extracellular acidification rate (ECAR) in PASMCs (n = 6) in vitro. Oligo: oligomycin; 2-DG: 2-deoxy-glucose. Data are shown as the mean ± SEM. P value is showed in each figure

To clarify whether CHCHD4 modulates mitochondrial function and energetics during hypoxic PAH, we firstly test ATP production in lung tissues after the Normoxia or hypoxia exposure for 4 weeks. Hypoxia-treated rats exhibited lower ATP levels than that in Normoxia controls at 28th day exposure, however, overexpression of CHCHD4 significantly improve ATP production under hypoxic condition (Fig. 5A). We next analyzed the morphology of mitochondria by transmission electron microscope (TEM). Intriguingly, Hypoxic PAH animals presented perceptible mitochondria engorge and cristae disappearance, compared to Normoxia controls, while exogenous CHCHD4 consequently compromised mitochondrial defects (Fig. 5B–D). In line with the results in vivo, mitochondrial swelling was also observed in isolated PASMCs under hypoxic condition, and CHCHD4 supplement antagonized hypoxia-induced abnormal mitochondrial morphology (Fig. 5E, F). Subsequently, to investigate how CHCHD4 is involved in hypoxia-mediated mitochondrial dysfunction, we measured mitochondrial oxidative phosphorylation and glycolysis using OCR and ECAR analysis, respectively. As expected, hypoxia-treated PASMCs exhibited decreased OCR and ECAR, however, CHCHD4 remarkably restored the mitochondrial function (Fig. 5G, H and Additional file 1: Fig. S4A, B). Thus, our findings demonstrated that CHCHD4 conspicuously antagonizes mitochondrial defects and dysfunction.

### CHCHD4 directly interacts with SAM50 to improve mitochondrial dynamics in PASMCs during hypoxic PAH

**Fig. 6:**
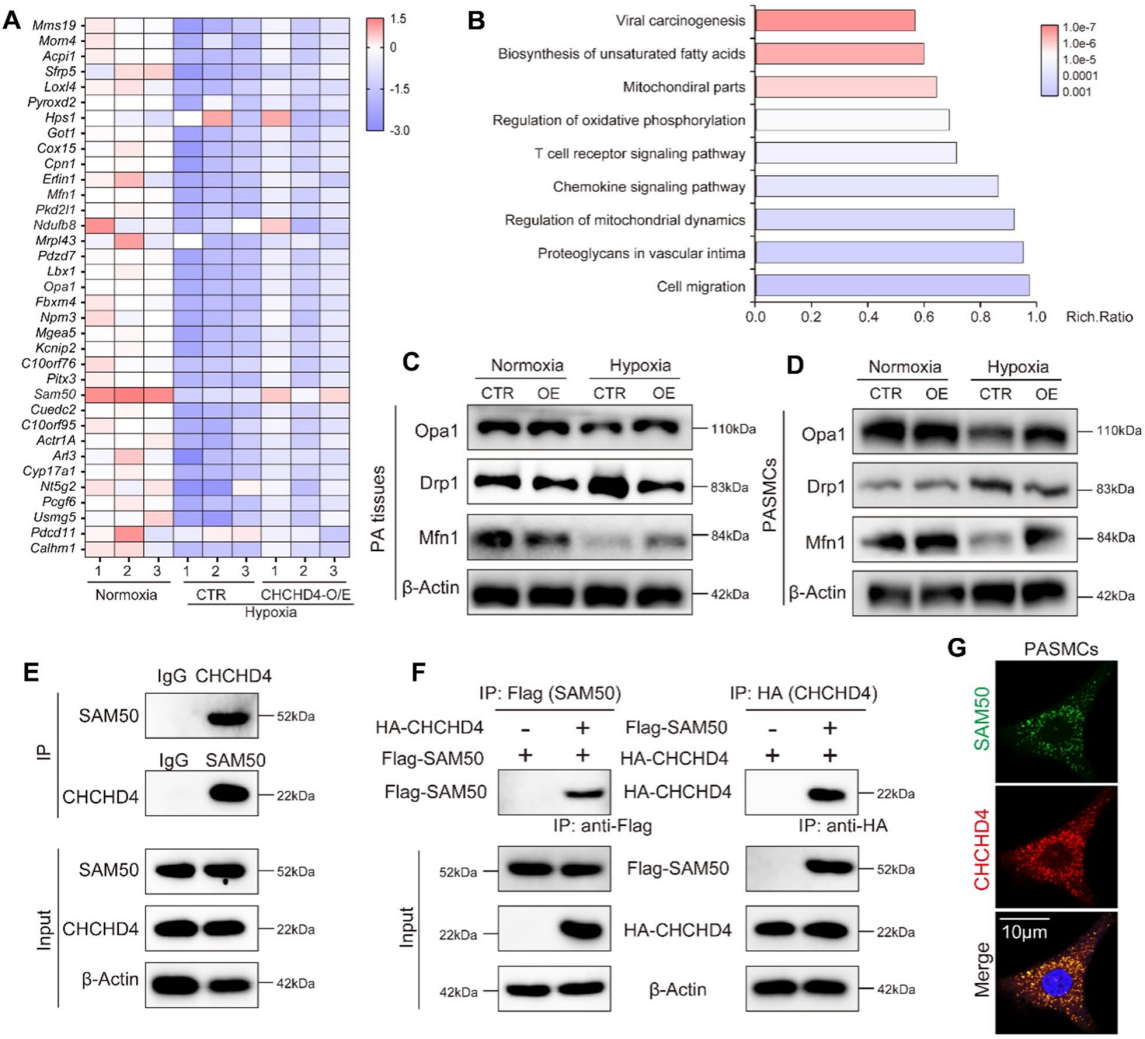
CHCHD4 modulates mitochondrial dynamics by binding to SAM50. **A** Hierarchical cluster heatmap of genes downregulated in hypoxia compared with Normoxia and upregulated with AAV-CHCHD4 injection in hypoxia. Data were collected for genes of interest filtered by a fold change > 1.2 and p < 0.05. We identified 35 proteins satisfying our criteria. **B** Pathway enrichment analysis of the 35 genes of interest. **C** Representative immunoblotting of Opa1, Drp1 and Mfn1 in lung PA tissues from indicated groups (n = 6). Normalized to β-Actin. **D** Representative immunoblotting of Opa1, Drp1 and Mfn1 in isolated PASMCs from indicated groups (n = 6). Normalized to β-Actin. **E** PASMCs lysates were immunoprecipitated with anti-CHCHD4 or SAM50 antibodies and probed with anti-SAM50 or anti-CHCHD4 antibodies (n = 3 independent experiments). **F** Flag-agarose beads or HA agarose were incubated with lysates of HEK 293 cells transfected with either Flag-tagged SAM50 or together with HA-tagged CHCHD4, and immunoprecipitation was probed with anti-HA or Flag antibody (n = 3 independent experiments). **G** Immunofluorescence images present SAM50 (green) and CHCHD4 (red) colocalization (merge, yellow) in PASMCs (n = 3 biological replicates)

To further investigate the potential mechanism of CHCHD4 during hypoxic PAH, we performed bulk RNA seq by using lung tissues from Normoxia or hypoxia treated animals with or without administration of AAV1-CHCHD4. 317 genes were downregulated, among which 35 genes were restored by CHCHD4 overexpression (Fig. 6A). And SAM50 expression significantly increased after AAV1-CHCHD4 treatment (Fig. 6A). Moreover, Pathway enrichment analysis showed that mitochondrial function-related genes were enriched (Fig. 6B). The optimal functioning of mitochondira in energy generations relies on proper mitochondrial dynamics, which includes fission and fusion processes [29, 32], in which SAM50 serves as a key regulator to maintain mitochondrial composition and OXPHOS [33, 34]. In addition, immunoblotting showed that overexpression of CHCHD4 remarkably antagonized hypoxia-induced reduction in Opa1 and Mfn1 expression, as well as impeded hypoxia-induced Drp1 upregulation in vivo (Fig. 6C and Additional file 1: Fig. S5A). In line with the data in vivo, PASMCs showed the same change trend (Fig. 6D and Additional file 1: Fig. S5B). Previously, SAM50 has been suggested to directly interact with CHCHD6 to sustain cristae structure [35]. To investigate the link between CHCHD4 and SAM50, we next performed immunoprecipitation assay and found that endogenous interaction of SAM50 and CHCHD4 was exhibited in PASMCs (Fig. 6E). Besides, exogenous immunoprecipitation assay demonstrated that SAM50 physically interact with CHCHD4 (Fig. 6F), and the confocal images showed SAM50 and CHCHD4 were co-localized together (Fig. 6G). In summary, our data show that CHCHD4 directly interacts with SAM50 to improve mitochondrial dynamics in PASMCs during hypoxic PAH.

### Disruption of SAM50 abrogates the beneficial effects of CHCHD4 in hypoxia-treated PASMCs

**Fig. 7:**
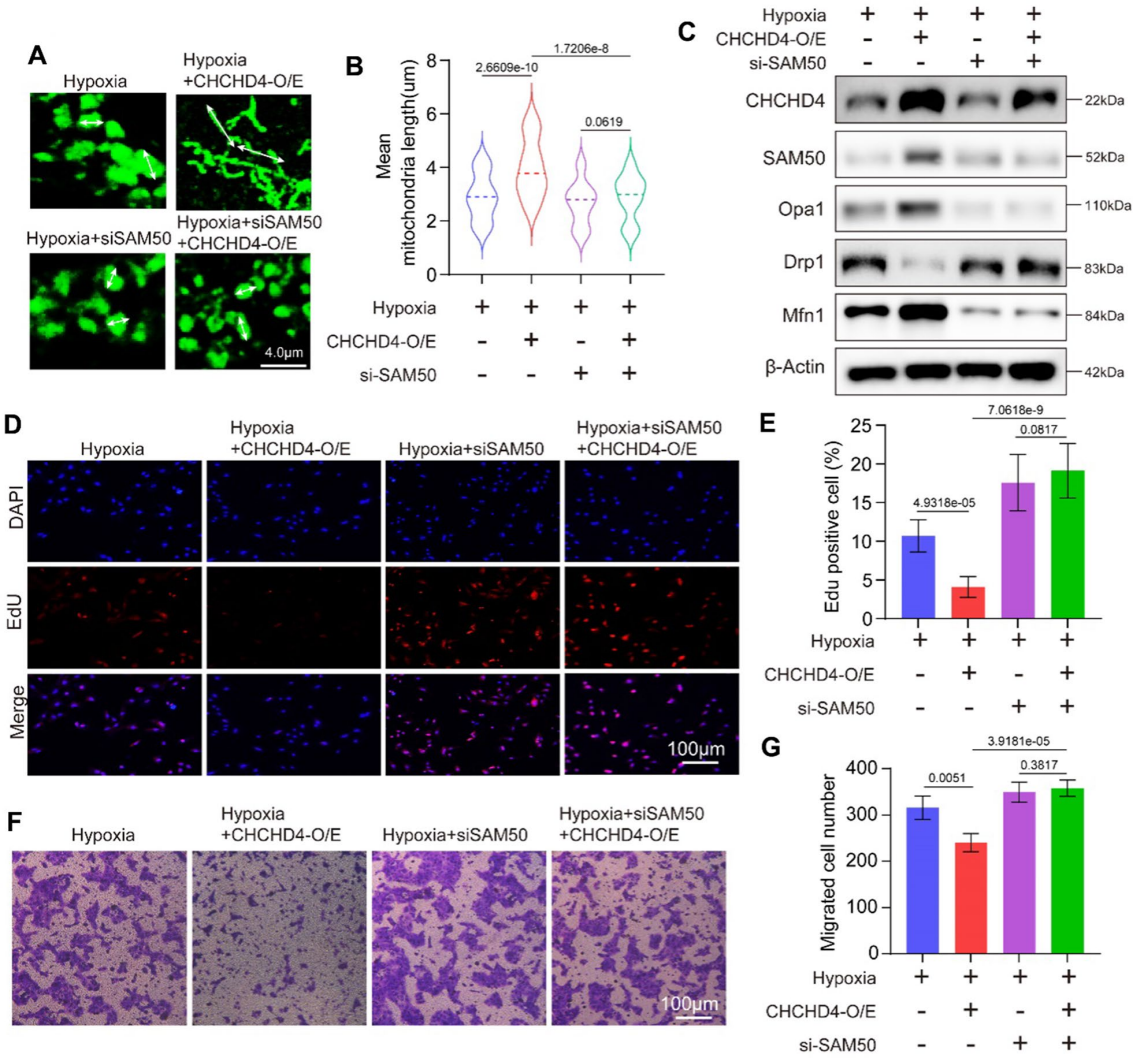
SAM50 knockdown abolishes the protective effects of CHHCD4 during hypoxia. **A**, **B** Representative confocal images of Mitochondrial Tracker in indicated group and quantification of the mitochondrial mean length (n = 73–108 PASMCs). White arow indicated the mitochondrial length. **C** Representative immunoblotting of SAM50, Opa1, Drp1 and Mfn1 in isolated PASMCs from indicated groups (n = 6). Normalized to β-Actin. **D**, **E** Representative images and summary data of the EdU incorporation assay. EdU positivity indicated that the cells were proliferating (n = 3 biological replicates). **F**, **G** Representative images and summary data of the Transwell assay, which indicated the migration ability of PASMCs in indicated groups (n = 3 independent experiments). Data are shown as the mean ± SEM. P value is showed in each figure

As SAM50 was identified as downstream effector target of CHCHD4, we next aimed to explore whether silencing SAM50 using siRNA could reverse the protective effects of CHCHD4 overexpression. The effects of CHCHD4-SAM50 crosstalk on mitochondria dynamics were also evaluated, and it was found that enhancing CHCHD4 gene expression significantly prevented mitochondrial swelling under hypoxic conditions, while downregulating SAM50 with siRNA promoted mitochondrial swelling during hypoxia, regardless of CHCHD4 overexpression (Fig. 7A, B). Likewise, knockdown of SAM50 abrogated CHCHD4 overexpression induced upregulation in Opa1 and Mfn1 expression, and reduction in Drp1 levels in response to hypoxia treatment, as identified by immunoblotting (Fig. 7C and Additional file 1: Fig. S6). Although enhancing CHCHD4 expression reduced the proliferation rate (Fig. 7D, E), and migration ability (Fig. 7F, G) when comparing to those in control group, loss of SAM50 significantly reverse the anti-proliferation and anti-migration effect of elevated CHCHD4 under hypoxia (Fig. 7D–G). In brief, our findings showed that CHCHD4 alleviated hypoxia-induced mitochondrial dysfunction, cell proliferation and migration in SAM50 dependent manner.

## Discussion

As a progressive, fatal cardiopulmonary disease, PAH is an insidious onset and rapid progression. The updated hemodynamic definition of PAH may improve case identification and treatment timing [36]. However, PAH is becoming more prevalent among elderly, with a more equal sex ratio and comorbidities, potentially leading to a less favorable therapeutic response [37]. Nonetheless, current clinical therapeutics for PH have not demonstrated clear benefit for patients. Therefore, deciphering the mechanisms and discovering new therapeutic targets were crucial for PAH treatment in clinical. Herein, we demonstrated that CHCHD4 orchestrates mitochondrial oxidative phosphorylation, and antagonizes aberrant proliferation and migration of PASMCs by directly interacting with SAM50, thereby disturbing hypoxic PAH, which could be a novel target for PAH treatment (Graph).

Proteins with CHCHD domains play crucial pathophysiological roles in mitochondria and other cellular processes. Mutation in these proteins have been linked to several human diseases, such as neurodegenerative diseases and tumors [9, 38]. Recent studies have shown strong correlation of CHCHD4 with poor prognosis in lung adenocarcinoma and development of tumor cell growth [21, 39]. Notably, CHCHD4 is found to affect redox regulation and mitochondrial intermembrane space (IMS) [40, 41].

The involvement of CHCHD4 and its interacting proteins in several common human diseases associated with mitochondrial dysfunction underscores the potential of CHCHD4 as a promising target for developing novel treatments for complex and devastating diseases. In current study, we uncovered that CHCHD4 promoted mitochondrial oxidative phosphorylation and blunted PASMCs’ aberrant proliferation and migration, by which CHCHD4 upregulation attenuated hypoxic PAH phenotype. SAM50 is a key component of the sorting and assembly machinery (SAM) complex responsible for biogenesis of β-barrel proteins [42]. It plays a vital role in mitochondrial intermembrane space bridging and biogenesis of respiratory complexes [43]. In addition, SAM50 has been reported to be directly interacted with CHCHD3 and CHCHD6 [35, 44], by which SAM50 signaling enhanced OPA1 and Mfn1/2 expression while downregulating p-Drp1 and Fis1. This results in the formation of tight and parallel cristae, augmented expression of cardiac mitochondrial complex subunits, increased ATP generation, but reduced secretion of cytochrome C from mitochondria and oxidative damage [44]. Disruption of SAM50 and CHCHD3 crosstalk triggered mitochondrial cristae remodeling and resulted in palmitate-induced cardiomyocyte hypertrophy [45]. Herein, we found that CHCHD4 directly bound to SAM50 in PASMCs to regulate mitochondrial oxidative phosphorylation and aberrant proliferation and migration of PASMCs in hypoxic PAH.

Abnormal mitochondrial dynamics, including fission, fusion, and autophagy, are a key pathological feature of PAH. These alterations contribute to unrestricted cell proliferation and apoptosis resistance, leading to the development of PAH. Downregulation of Mfn2 impairs mitochondrial fusion, while excessive activation of Drp1 triggers aberrant mitochondrial fission, both of which are elevated in PAH patients [46–50]. Dysfunctional mitochondrial autophagy has been reported to generate excessive amount of reactive oxygen species (ROS), and it is also involved in modulating PAH by regulating AMPK/mTOR signal pathway [51, 52]. In current study, we found that CHCHD4 overexpression contributed to Mfn1 and Opa1 expression, and suppressed hypoxia induced Drp1 upregulation. We revealed that CHCHD4 modulated mitochondrial dynamics by directly interacting with SAM50, therefore targeting defective mitochondrial dynamics might serve as a potential approach for PAH treatment.

### Limitations

Although we uncovered that CHCHD4 significantly restored mitochondrial oxidative phosphorylation in PASMCs, the link between defective mitochondria and proliferation and migration of PASMCs deserved further research. Moreover, we provide a new insight for CHCHD4-mediated protective effects in PAH, however, whether and how other CHCHDs regulates PAH progression is poorly understood. Here, male adult rats are utilized in our exploration, and the deviation attributed to sex difference should not be ignored. Importantly, insufficient evidence of CHCHD4 agonist hinders the clinical and translational value in this research.

## Conclusion

We revealed that CHCHD4 was significantly downregulated among CHCHD proteins in rat PASMCs during hypoxia and lung tissues of rats with hypoxia-induced PAH. Interestingly, AAV serotype 1-induced CHCHD4 elevation conspicuously alleviates pulmonary artery resistance and vascular remodeling, and orchestrates mitochondrial oxidative phosphorylation in PASMCs. We also found overexpression of CHCHD4 impeded proliferation and migration of PASMCs. Mechanistically, through lung tissues bulk RNA-sequencing (RNA-seq), we further identified CHCHD4 modulated mitochondrial dynamics by directly interacting with SAM50, a barrel protein on mitochondrial outer membrane surface. Furthermore, knockdown of SAM50 reversed the biological effects of CHCHD4 overexpression in isolated PASMCs. We demonstrated that elevated CHCHD4 orchestrates mitochondrial oxidative phosphorylation and antagonizes aberrant proliferation and migration of PASMCs, thereby disturbing hypoxic PAH.

## Supplementary Information

**Additional file 1: Figure S1.** CHCHD4 is identified as a regulator of PAH. Related to Fig. 1. **A** Quantification of ratio of pulmonary arterial medial thickness to total vessel size (media/CSA) in indicated group (n = 6). Related to Fig. 1D. **B** Quantification of immunoblotting of CHCHD4 (n = 6). Related to Fig. 1C. **C** Quantitative analysis of α-SMA and CHCHD4 fluorescence intensity by Image J software (n=6). Related to Fig. 1E. **D** Representative immunofluorescence of Vimentin (green), CHCHD4 (red) and DAPI (blue) in lung tissues from indicated group. And quantitative analysis of CHCHD4 fluorescence intensity by Image J software (n = 6). Data are shown as the mean ± SEM. P value is showed in each figure. **Figure S2.** Protocols. **A** Experimental schedule of SD rats received AAV1-Chchd4 or CTR injection. Representative immunoblotting of CHCHD4 in lung tissues from animals with AAV1-CTR or Chchd4. Related to Fig. 3. **B** VSMCs, ECs and other cells were isolated from SD rats. Related to Fig. 1. **C** Experimental schedule of primary PASMCs received Chchd4 overexpression or knockdown in vitro. Related to Fig. 4. **Figure S3.** CHCHD4 affects hypoxia-induced migration in PASMCs. **A** The percent of wound closure of PASMCs received len-CTR or len-CHCHD4 transduction was counted (n = 6 fields from 3 independent experiments). Related to Fig. 4B. **B** The percent of wound closure of PASMCs received siCTR or siCHCHD4 transduction was counted (n = 6 fields from 3 independent experiments). Related to Fig. 4G. Data are shown as the mean ± SEM. P value is showed in each figure. **Figure S4.** Overexpression of CHCHD4 improves hypoxia-induced mitochondrial dysfunction. Related to Fig. 5. **A** Quantification of basal respiration, ATP production–coupled respiration, maximal respiration, and spare respiratory capacity from oxygen consumption rate (OCR) in PASMCs. Related to Fig. 5G. **B** Quantification of glycolysis, glycolytic capacity and glycolytic reserve from extracellular acidification rate (ECAR) in PASMCs. Related to Fig. 5H. Data are shown as the mean ± SEM. P value is showed in each figure. **Figure S5.** CHCHD4 modulates mitochondrial dynamics. Related to Fig. 6. **A**. Quantification of immunoblotting of Opa1, Drp1 and Mfn1 in lung PA tissues from indicated groups (n = 6). Related to Fig. 6C. **B** Quantification of immunoblotting of Opa1, Drp1 and Mfn1 in isolated PASMCs from indicated groups (n = 6). Related to Fig. 6D. Data are shown as the mean ± SEM. P value is showed in each figure. **Figure S6.** SAM50 knockdown abolishes the protective effects of CHHCD4 during hypoxia. Related to Fig. 7. Quantification of immunoblotting of CHCHD4, SAM50, Opa1, Drp1 and Mfn1 in isolated PASMCs from indicated groups (n = 6). Related to Fig. 7C.

## Data Availability

The datasets used and analyzed during the current study are available from the corresponding author on reasonable request.

## Abbreviations

AAV1: Serotype 1 adeno‐associated viral vector
α-SMA: Alpha smooth muscle Actin
CHCHD4: Coiled–coil–helix–coiled–coil–helix domain-containing protein 4
CTR: Control
OE: Overexpression
OXPHOS: Oxidative phosphorylation
PAH: Pulmonary arterial hypertension
PASMCs: Pulmonary artery smooth muscle cells of rats
PCNA: Proliferating cell nuclear antigen
RVSP: Right ventricular systolic pressure

## References

[1] Singh N, Dorfmuller P, Shlobin OA, Ventetuolo CE (2022). Group 3 pulmonary hypertension: from bench to bedside. Circ Res.

[2] Naeije R, Richter MJ, Rubin LJ (2022). The physiological basis of pulmonary arterial hypertension. Eur Respir J.

[3] Zheng Y (2023). Deficiency of filamin A in smooth muscle cells protects against hypoxia-mediated pulmonary hypertension in mice. Int J Mol Med.

[4] Chu C (2023). Swietenine alleviates vascular remodeling by enhancing mitophagy of pulmonary arterial smooth muscle cells in experimental pulmonary hypertension. Can J Cardiol.

[5] Cuthbertson I, Morrell NW, Caruso P (2023). BMPR2 mutation and metabolic reprogramming in pulmonary arterial hypertension. Circ Res.

[6] Wertheim BM (2023). Proline and glucose metabolic reprogramming supports vascular endothelial and medial biomass in pulmonary arterial hypertension. JCI Insight.

[7] Chen J (2022). Sphingosine kinase 1 deficiency in smooth muscle cells protects against hypoxia-mediated pulmonary hypertension via YAP1 signaling. Int J Mol Sci.

[8] Gluschke H (2022). Autoimmunity to sphingosine-1-phosphate-receptors in systemic sclerosis and pulmonary arterial hypertension. Front Immunol.

[9] Zhou ZD, Saw WT, Tan EK (2017). Mitochondrial CHCHD-containing proteins: physiologic functions and link with neurodegenerative diseases. Mol Neurobiol.

[10] Fan L (2022). CHCHD2 p.Thr61Ile knock-in mice exhibit motor defects and neuropathological features of Parkinson's disease. Brain Pathol.

[11] Lu L (2022). CHCHD2 maintains mitochondrial contact site and cristae organizing system stability and protects against mitochondrial dysfunction in an experimental model of Parkinson's disease. Chin Med J (Engl).

[12] Liu W (2020). Chchd2 regulates mitochondrial morphology by modulating the levels of Opa1. Cell Death Differ.

[13] Zhou W, Ma D, Tan EK (2020). Mitochondrial CHCHD2 and CHCHD10: roles in neurological diseases and therapeutic implications. Neuroscientist.

[14] Li Y (2022). Increased CHCHD2 expression promotes liver fibrosis in nonalcoholic steatohepatitis via Notch/osteopontin signaling. JCI Insight..

[15] Xue X (2022). Tumour cells are sensitised to ferroptosis via RB1CC1-mediated transcriptional reprogramming. Clin Transl Med.

[16] Jiang T, Wang Y, Wang X, Xu J (2022). CHCHD2 and CHCHD10: Future therapeutic targets in cognitive disorder and motor neuron disorder. Front Neurosci.

[17] Xia W (2022). Chchd10 is dispensable for myogenesis but critical for adipose browning. Cell Regen.

[18] Ding M (2022). CHCHD10 modulates thermogenesis of adipocytes by regulating lipolysis. Diabetes.

[19] Petrungaro C (2015). The Ca(2+)-dependent release of the Mia40-induced MICU1-MICU2 dimer from MCU regulates mitochondrial Ca(2+) uptake. Cell Metab.

[20] Al-Habib H, Ashcroft M (2021). CHCHD4 (MIA40) and the mitochondrial disulfide relay system. Biochem Soc Trans.

[21] Thomas LW (2019). CHCHD4 regulates tumour proliferation and EMT-related phenotypes, through respiratory chain-mediated metabolism. Cancer Metab.

[22] Thomas LW (2019). CHCHD4 confers metabolic vulnerabilities to tumour cells through its control of the mitochondrial respiratory chain. Cancer Metab.

[23] Wang T (2021). C9orf72 regulates energy homeostasis by stabilizing mitochondrial complex I assembly. Cell Metab.

[24] Yang J (2012). Human CHCHD4 mitochondrial proteins regulate cellular oxygen consumption rate and metabolism and provide a critical role in hypoxia signaling and tumor progression. J Clin Invest.

[25] Whitley BN, Engelhart EA, Hoppins S (2019). Mitochondrial dynamics and their potential as a therapeutic target. Mitochondrion.

[26] Wai T, Langer T (2016). Mitochondrial dynamics and metabolic regulation. Trends Endocrinol Metab.

[27] Hong X (2022). Mitochondrial dynamics maintain muscle stem cell regenerative competence throughout adult life by regulating metabolism and mitophagy. Cell Stem Cell.

[28] Kleele T (2021). Distinct fission signatures predict mitochondrial degradation or biogenesis. Nature.

[29] Chan DC (2020). Mitochondrial dynamics and its involvement in disease. Annu Rev Pathol.

[30] Xie S (2022). Long-term activation of glucagon-like peptide-1 receptor by dulaglutide prevents diabetic heart failure and metabolic remodeling in type 2 diabetes. J Am Heart Assoc.

[31] Mura M, Cecchini MJ, Joseph M, Granton JT (2019). Osteopontin lung gene expression is a marker of disease severity in pulmonary arterial hypertension. Respirology.

[32] Giacomello M, Pyakurel A, Glytsou C, Scorrano L (2020). The cell biology of mitochondrial membrane dynamics. Nat Rev Mol Cell Biol.

[33] Li X (2022). Mitochondria shed their outer membrane in response to infection-induced stress. Science.

[34] Takeda H (2021). Mitochondrial sorting and assembly machinery operates by beta-barrel switching. Nature.

[35] Ding C (2015). Mitofilin and CHCHD6 physically interact with Sam50 to sustain cristae structure. Sci Rep.

[36] Anderson JJ, Lau EM (2022). Pulmonary hypertension definition, classification, and epidemiology in Asia. JACC Asia.

[37] Hoeper MM (2013). Elderly patients diagnosed with idiopathic pulmonary arterial hypertension: results from the COMPERA registry. Int J Cardiol.

[38] Liu T (2022). Modulation of synaptic plasticity, motor unit physiology, and TDP-43 pathology by CHCHD10. Acta Neuropathol Commun.

[39] Zhou Y (2022). USF1-CHCHD4 axis promotes lung adenocarcinoma progression partially via activating the MYC pathway. Discov Oncol.

[40] Dickson-Murray E, Nedara K, Modjtahedi N, Tokatlidis K (2021). The Mia40/CHCHD4 oxidative folding system: redox regulation and signaling in the mitochondrial intermembrane space. Antioxidants (Basel).

[41] Thomas LW, Staples O, Turmaine M, Ashcroft M (2017). CHCHD4 regulates intracellular oxygenation and perinuclear distribution of mitochondria. Front Oncol.

[42] Lionello S, Marzaro G, Martinvalet D (2020). SAM50, a side door to the mitochondria: the case of cytotoxic proteases. Pharmacol Res.

[43] Ott C (2012). Sam50 functions in mitochondrial intermembrane space bridging and biogenesis of respiratory complexes. Mol Cell Biol.

[44] Xue RQ (2019). Pyridostigmine alleviates cardiac dysfunction via improving mitochondrial cristae shape in a mouse model of metabolic syndrome. Free Radic Biol Med.

[45] Xue RQ (2019). Regulation of mitochondrial cristae remodelling by acetylcholine alleviates palmitate-induced cardiomyocyte hypertrophy. Free Radic Biol Med.

[46] Xiao F, Zhang R, Wang L (2022). Inhibitors of mitochondrial dynamics mediated by dynamin-related protein 1 in pulmonary arterial hypertension. Front Cell Dev Biol.

[47] Dasgupta A (2021). PINK1-induced phosphorylation of mitofusin 2 at serine 442 causes its proteasomal degradation and promotes cell proliferation in lung cancer and pulmonary arterial hypertension. FASEB J.

[48] Ryan JJ (2013). PGC1alpha-mediated mitofusin-2 deficiency in female rats and humans with pulmonary arterial hypertension. Am J Respir Crit Care Med.

[49] Feng W (2021). ERK/Drp1-dependent mitochondrial fission contributes to HMGB1-induced autophagy in pulmonary arterial hypertension. Cell Prolif.

[50] Tian L (2018). Increased Drp1-mediated mitochondrial fission promotes proliferation and collagen production by right ventricular fibroblasts in experimental pulmonary arterial hypertension. Front Physiol.

[51] Ornatowski W (2020). Complex interplay between autophagy and oxidative stress in the development of pulmonary disease. Redox Biol.

[52] Wei R (2022). Silencing TUFM inhibits development of monocrotaline-induced pulmonary hypertension by regulating mitochondrial autophagy via AMPK/mTOR signal pathway. Oxid Med Cell Longev.

