# Supplementary material for: Elevated CHCHD4 orchestrates mitochondrial oxidative phosphorylation to disturb hypoxic pulmonary hypertension
Source: J Transl Med. 2023 Jul 12;21:464. doi: 10.1186/s12967-023-04268-3 (PMC10339524; doi:10.1186/s12967-023-04268-3)
Supplement: Supplementary file 1 — Additional file 1: Figure S1. CHCHD4 is identified as a regulator of PAH. Related to Fig. 1. A Quantification of ratio of pulmonary arterial medial thickness to total vessel size (media/CSA) in indicated group (n = 6). Related to Fig. 1D. B Quantification of immunoblotting of CHCHD4 (n = 6). Related to Fig. 1C. C Quantitative analysis of α-SMA and CHCHD4 fluorescence intensity by Image J software (n=6). Related to Fig. 1E. D Representative immunofluorescence of Vimentin (green), CHCHD4 (red) and DAPI (blue) in lung tissues from indicated group. And quantitative analysis of CHCHD4 fluorescence intensity by Image J software (n = 6). Data are shown as the mean ± SEM. P value is showed in each figure. Figure S2. Protocols. A Experimental schedule of SD rats received AAV1-Chchd4 or CTR injection. Representative immunoblotting of CHCHD4 in lung tissues from animals with AAV1-CTR or Chchd4. Related to Fig. 3. B VSMCs, ECs and other cells were isolated from SD rats. Related to Fig. 1. C Experimental schedule of primary PASMCs received Chchd4 overexpression or knockdown in vitro. Related to Fig. 4. Figure S3. CHCHD4 affects hypoxia-induced migration in PASMCs. A The percent of wound closure of PASMCs received len-CTR or len-CHCHD4 transduction was counted (n = 6 fields from 3 independent experiments). Related to Fig. 4B. B The percent of wound closure of PASMCs received siCTR or siCHCHD4 transduction was counted (n = 6 fields from 3 independent experiments). Related to Fig. 4G. Data are shown as the mean ± SEM. P value is showed in each figure. Figure S4. Overexpression of CHCHD4 improves hypoxia-induced mitochondrial dysfunction. Related to Fig. 5. A Quantification of basal respiration, ATP production–coupled respiration, maximal respiration, and spare respiratory capacity from oxygen consumption rate (OCR) in PASMCs. Related to Fig. 5G. B Quantification of glycolysis, glycolytic capacity and glycolytic reserve from extracellular acidification rate (ECAR) in PASMCs. Related [file 12967_2023_4268_MOESM1_ESM.docx]

**Additional file 1: Materials**


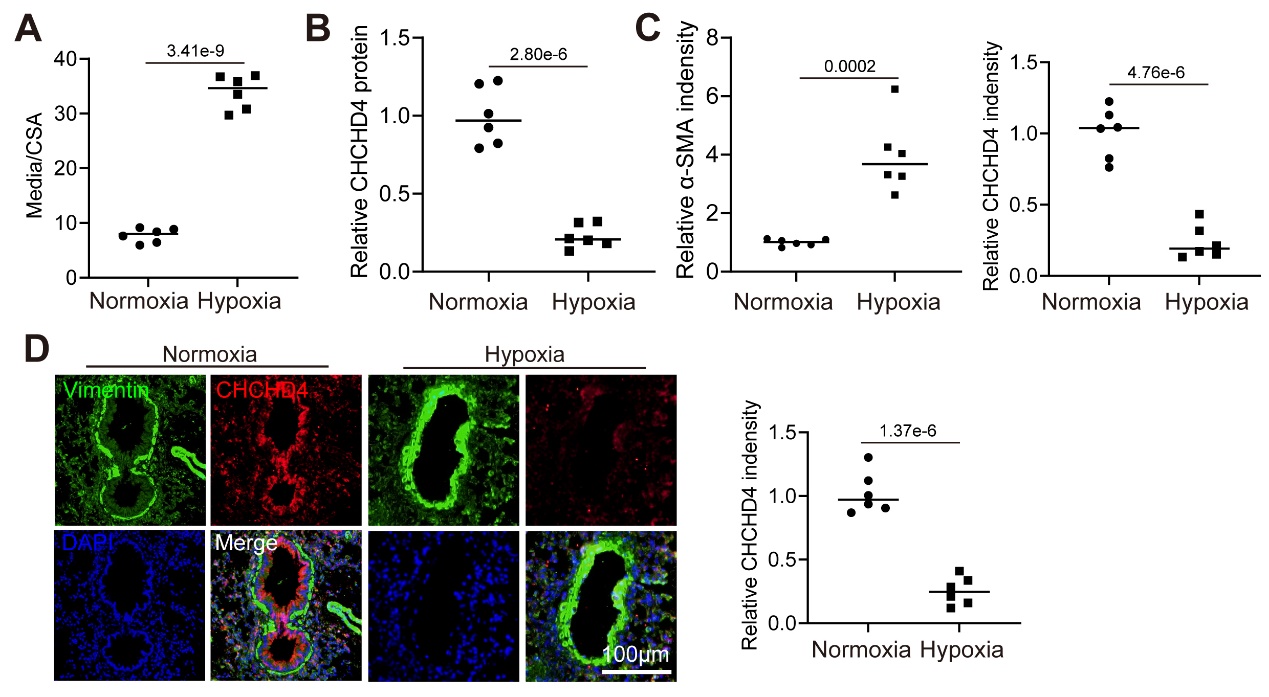


**Figure S1.** **CHCHD4 is identified as a regulator of PAH.**

**Related to figure 1.**

**A.** Quantification of ratio of pulmonary arterial medial thickness to total vessel size (media/CSA) in indicated group (n=6). **Related to figure 1D.**

**B.** Quantification of immunoblotting of CHCHD4 (n=6). **Related to figure 1C.**

**C.** Quantitative analysis of α-SMA and CHCHD4 fluorescence intensity by Image J software (n=6). **Related to figure 1E.**

**D.** Representative immunofluorescence of Vimentin (green), CHCHD4 (red) and DAPI (blue) in lung tissues from indicated group. And quantitative analysis of CHCHD4 fluorescence intensity by Image J software (n=6).

Data are shown as the mean±SEM. P value is showed in each figure.


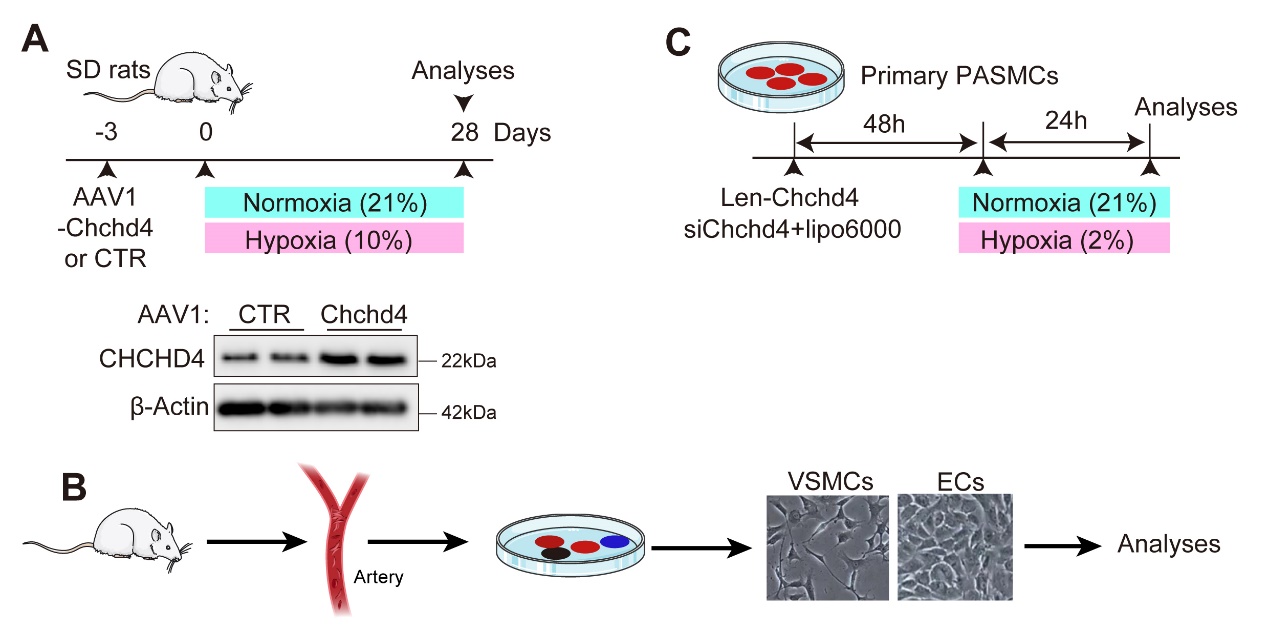


**Figure S2.** **Protocols.**

**A.** Experimental schedule of SD rats received AAV1-Chchd4 or CTR injection. Representative immunoblotting of CHCHD4 in lung tissues from animals with AAV1-CTR or Chchd4. **Related to figure 3.**

**B.** VSMCs, ECs and other cells were isolated from SD rats. **Related to Figure 1.**

**C.** Experimental schedule of primary PASMCs received Chchd4 overexpression or knockdown in vitro. **Related to figure 4.**


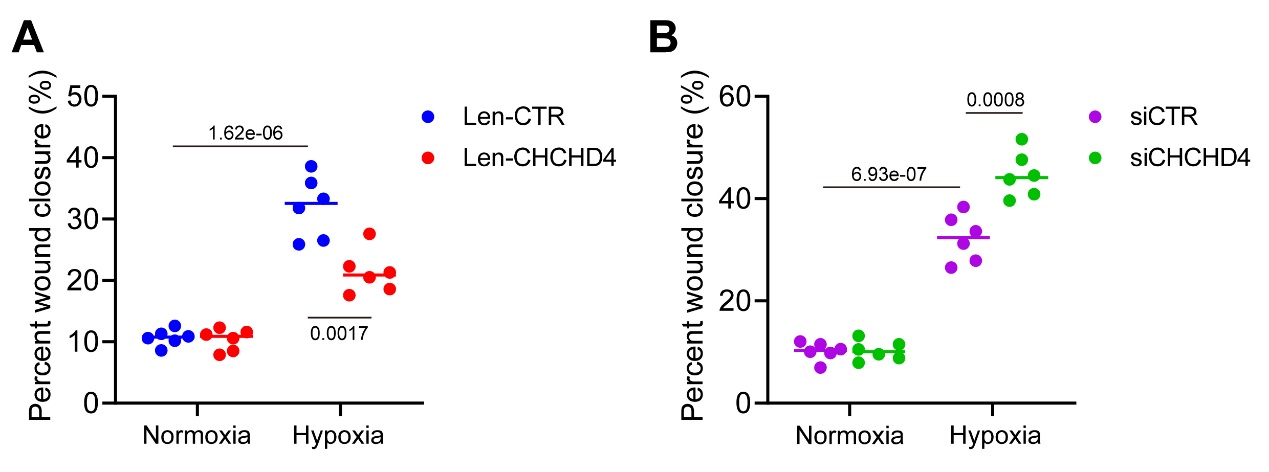


**Figure S3. CHCHD4 affects hypoxia-induced migration in PASMCs.**

**A.** The percent of wound closure of PASMCs received len-CTR or len-CHCHD4 transduction was counted (n=6 fields from 3 independent experiments). **Related to figure 4B.**

**B.** The percent of wound closure of PASMCs received siCTR or siCHCHD4 transduction was counted (n=6 fields from 3 independent experiments). **Related to figure 4G.**

Data are shown as the mean±SEM. P value is showed in each figure.


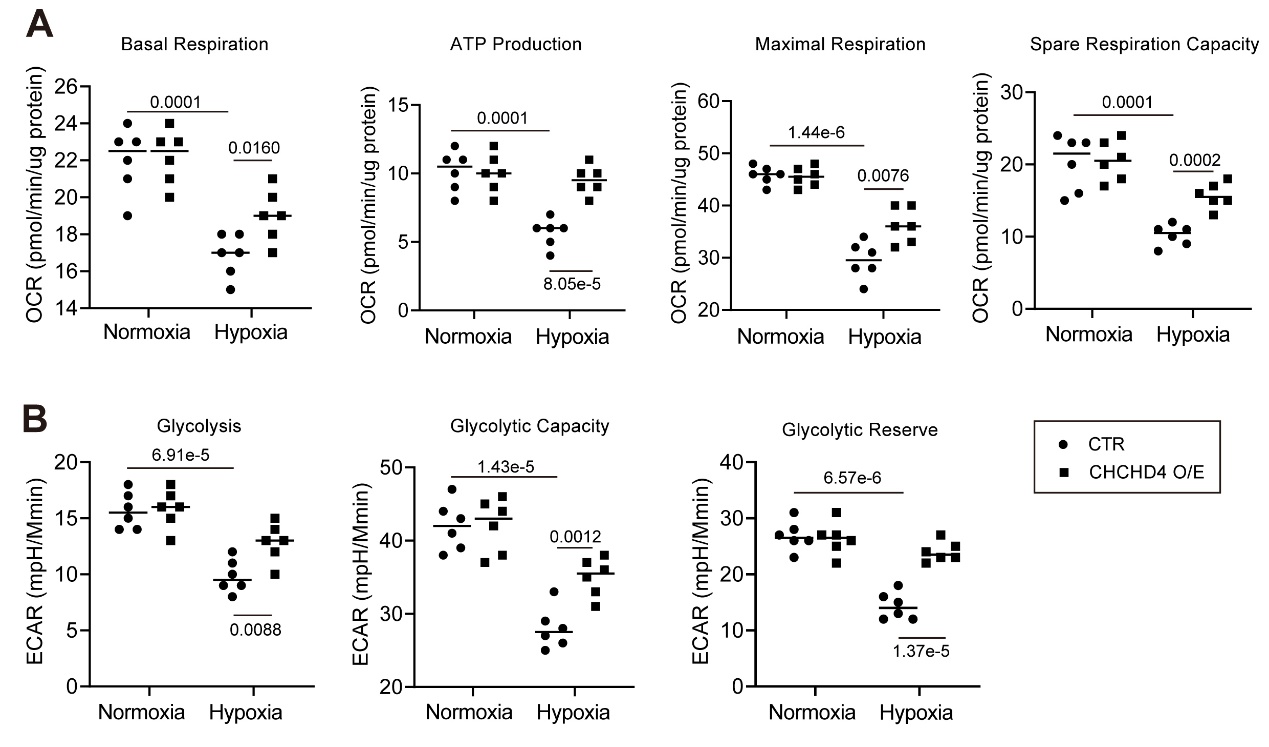


**Figure S4. Overexpression of CHCHD4 improves hypoxia-induced mitochondrial dysfunction. Related to figure 5.**

**A.** Quantification of basal respiration, ATP production–coupled respiration, maximal respiration, and spare respiratory capacity from oxygen consumption rate (OCR) in PASMCs. **Related to figure 5G.**

**B.** Quantification of glycolysis, glycolytic capacity and glycolytic reserve from extracellular acidification rate (ECAR) in PASMCs. **Related to figure 5H.**

Data are shown as the mean±SEM. P value is showed in each figure.


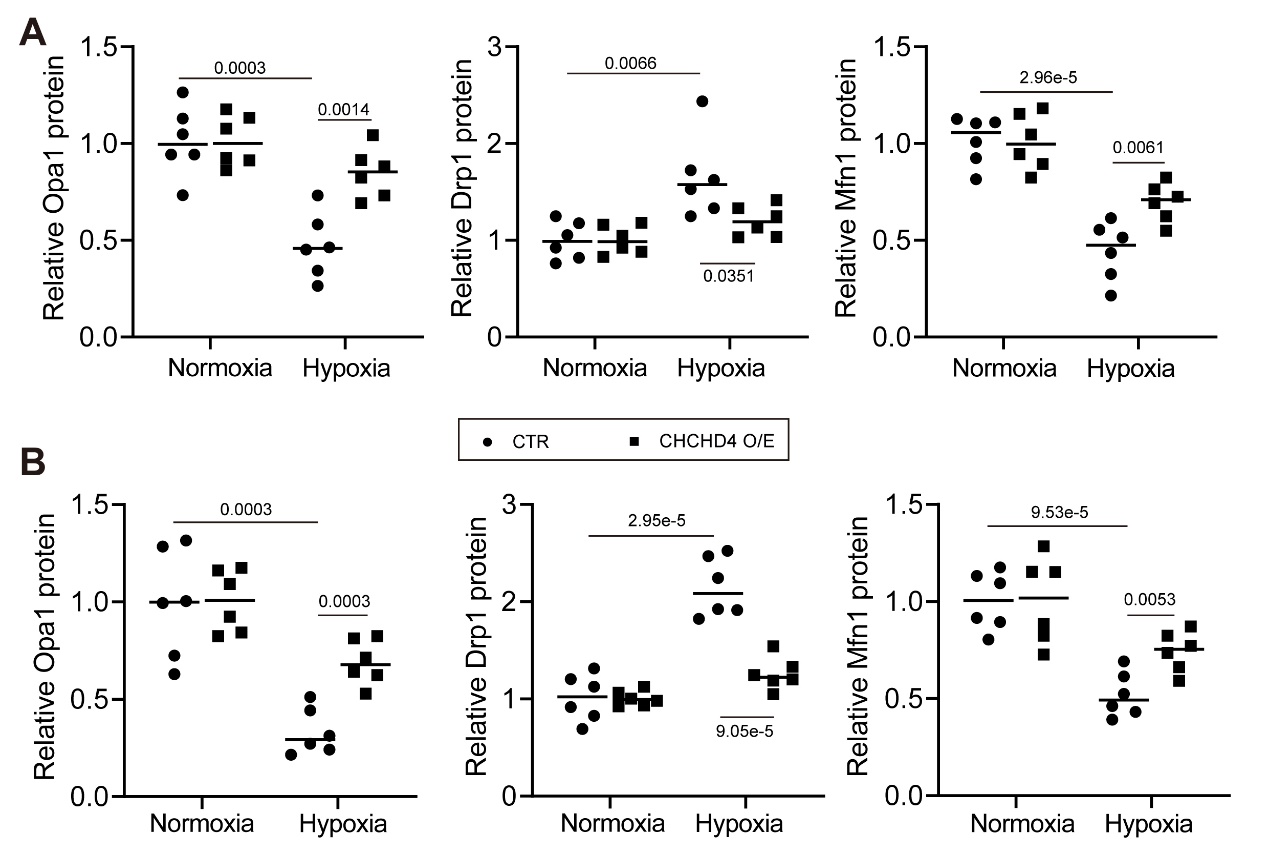


**Figure S5. CHCHD4 modulates mitochondrial dynamics.** **Related to figure 6.**

**A.** Quantification of immunoblotting of Opa1, Drp1 and Mfn1 in lung PA tissues from indicated groups (n=6). **Related to figure 6C.**

**B.** Quantification of immunoblotting of Opa1, Drp1 and Mfn1 in isolated PASMCs from indicated groups (n=6). **Related to figure 6D.**

Data are shown as the mean±SEM. P value is showed in each figure.


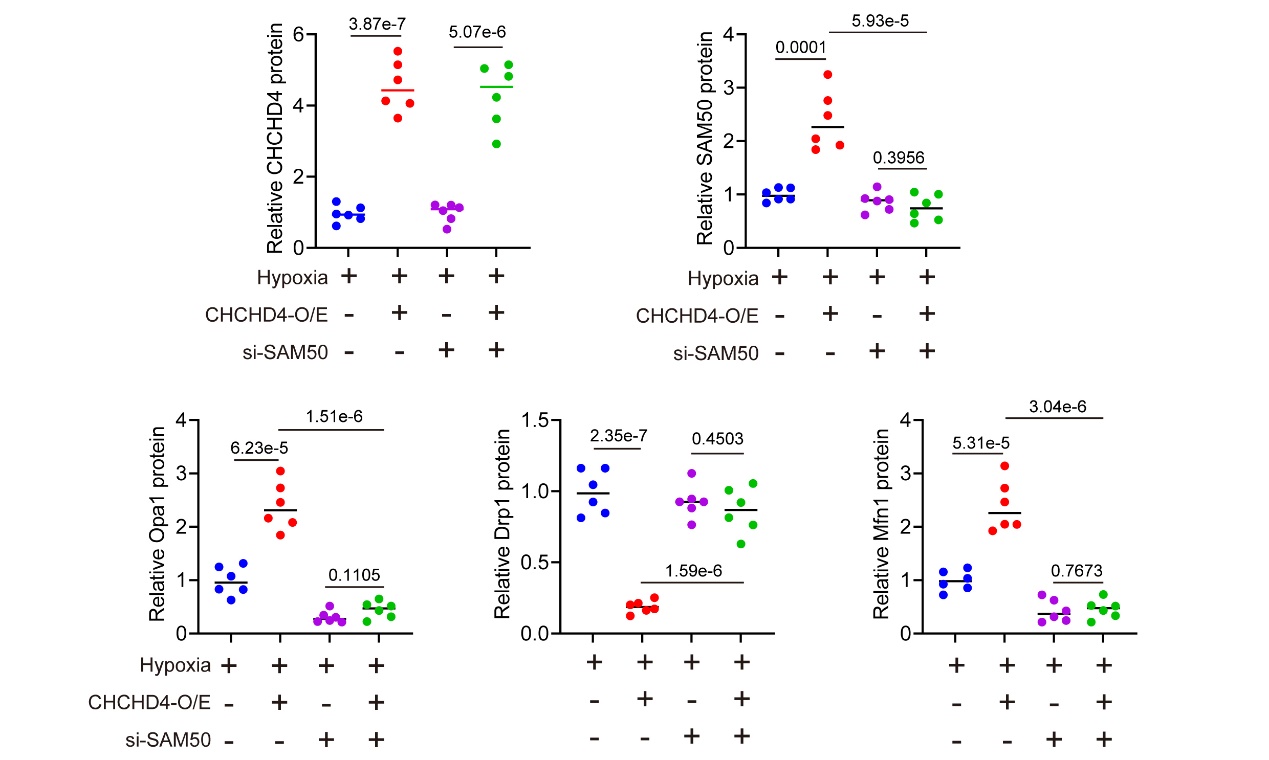


**Figure S6.** **SAM50 knockdown abolishes the protective effects of CHHCD4 during hypoxia. Related to figure 7.**

Quantification of immunoblotting of CHCHD4, SAM50, Opa1, Drp1 and Mfn1 in isolated PASMCs from indicated groups (n=6). **Related to figure 7C.**
